# Regulation of Hippocampal 5-HT Release by P2X7 Receptors in Response to Optogenetic Stimulation of Median Raphe Terminals of Mice

**DOI:** 10.3389/fnmol.2017.00325

**Published:** 2017-10-12

**Authors:** Flóra Gölöncsér, Mária Baranyi, Diána Balázsfi, Kornél Demeter, József Haller, Tamás F. F. Freund, Dóra Zelena, Beáta Sperlágh

**Affiliations:** ^1^Laboratory of Molecular Pharmacology, Institute of Experimental Medicine, Hungarian Academy of Sciences, Budapest, Hungary; ^2^János Szentágothai School of Neurosciences, Semmelweis University School of Ph.D. Studies, Budapest, Hungary; ^3^Laboratory of Stress and Behavior Neurobiology, Institute of Experimental Medicine, Hungarian Academy of Sciences, Budapest, Hungary; ^4^Unit of Behavioral Studies, Institute of Experimental Medicine, Hungarian Academy of Sciences, Budapest, Hungary; ^5^Laboratory of Cerebral Cortex, Institute of Experimental Medicine, Hungarian Academy of Sciences, Budapest, Hungary

**Keywords:** serotonin, neurotransmitter release, optogenetics, median raphe nucleus, hippocampus, P2X7 receptor, JNJ-47965567, AZ-10606120

## Abstract

Serotonergic and glutamatergic neurons of median raphe region (MRR) play a pivotal role in the modulation of affective and cognitive functions. These neurons synapse both onto themselves and remote cortical areas. P2X7 receptors (*P2rx7*) are ligand gated ion channels expressed by central presynaptic excitatory nerve terminals and involved in the regulation of neurotransmitter release. *P2rx7*s are implicated in various neuropsychiatric conditions such as schizophrenia and depression. Here we investigated whether 5-HT release released from the hippocampal terminals of MRR is subject to modulation by *P2rx7*s. To achieve this goal, an optogenetic approach was used to selectively activate subpopulation of serotonergic terminals derived from the MRR locally, and one of its target area, the hippocampus. Optogenetic activation of neurons in the MRR with 20 Hz was correlated with freezing and enhanced locomotor activity of freely moving mice and elevated extracellular levels of 5-HT, glutamate but not GABA *in vivo*. Similar optical stimulation (OS) significantly increased [^3^H]5-HT and [^3^H]glutamate release in acute MRR and hippocampal slices. We examined spatial and temporal patterns of [^3^H]5-HT release and the interaction between the serotonin and glutamate systems. Whilst [^3^H]5-HT release from MRR neurons was [Ca^2+^]_o_-dependent and sensitive to TTX, CNQX and DL-AP-5, release from hippocampal terminals was not affected by the latter drugs. Hippocampal [^3^H]5-HT released by electrical but not OS was subject to modulation by 5- HT1_B/D_ receptors agonist sumatriptan (1 μM), whereas the selective 5-HT_1A_ agonist buspirone (0.1 μM) was without effect. [^3^H]5-HT released by electrical and optical stimulation was decreased in mice genetically deficient in *P2rx7*s, and after perfusion with selective *P2rx7* antagonists, JNJ-47965567 (0.1 μM), and AZ-10606120 (0.1 μM). Optical and electrical stimulation elevated the extracellular level of ATP. Our results demonstrate for the first time the modulation of 5-HT release from hippocampal MRR terminals by the endogenous activation of *P2rx7*s. *P2rx7* mediated modulation of 5-HT release could contribute to various physiological and pathophysiological phenomena, related to hippocampal serotonergic transmission.

## Introduction

Behaviors are associated with different operational modes of the nervous system under the control of both the autonomic/internal state of the animal and external influences. The organizational principle, i.e., the extreme divergence of projections, enables subcortical modulatory centers to synchronously affect, and thereby functionally couple distinct brain regions (Vertes et al., 1999). Based on the temporal scale of influence, subcortical modulation can be divided into a slow, tonic and a faster, phasic form (Sarter et al., 2009; Zweifel et al., 2009). The prominent example of the parallel operation of slow, tonic and fast, phasic modulation has been detected in the ascending serotonergic system. Thin axons of DR serotonergic neurons are studded with small varicosities and rarely establish synapses, whereas median raphe (MR) serotonergic cells give rise to thick axons with large varicosities, most of which make synaptic contacts on cortical GABAergic interneurons (Mulligan and Tork, 1988; Freund et al., 1990; Wilson and Molliver, 1991). Consequently, the DR projection would be ideal for engulfing large areas by volume transmission, whereas the MR may employ both the diffuse non-synaptic, as well as the faster, target-specific, phasic modulation. Optogenetic activation of DR and MR nuclei revealed a unexpectedly fast component of MR-hippocampal activation (Varga et al., 2009) and the involvement of these nuclei in shaping of different behaviors, such as anxiety (Ohmura et al., 2014), sleep regulation (Ito et al., 2013) and memory consolidation (Wang et al., 2015). These data suggest that subpopulations of DR and MR have distinct implications in physiological function and behavior.

P2X7 receptors are ligand-gated ion channels activated by high concentration of extracellular ATP. In the central nervous system, *P2rx7*s are expressed by both neuronal and non-neuronal cells and involved in a variety of physiopathological functions including the modulation of neurotransmitter release, the regulation of the production of pro-inflammatory cytokines and the microglia mediated neuronal death (Sperlagh and Illes, 2014). The activation of *P2rx7*s elicit glutamate and subsequent GABA release from the HP (Sperlagh et al., 2002), but also mediates the release of the endogenous *P2rx7* agonist ATP (Heinrich et al., 2012). *P2rx7*s have been shown to contribute to the pathophysiology of depression (Csolle et al., 2013a,b; Iwata et al., 2016; Otrokocsi et al., 2017), bipolar disorder (Csolle et al., 2013a; Lord et al., 2014) and schizophrenia (Kovanyi et al., 2016). However, the regulation of serotonergic transmission by *P2rx7*s have been remained controversial so far. Using mice genetically deficient in *P2rx7*s an elevated basal 5-HT level was found in the HP, with a lower turnover of 5-HT and increased number of [^3^H]citalopram binding sites indicating the tonic impact of *P2rx7* activity to serotonergic transmission (Csolle et al., 2013b). Further, the stress-induced elevation of 5-HT level was also alleviated in the absence of *P2rx7*s (Csolle et al., 2013b). In another study, JNJ-42253432, the *P2rx7* antagonist increased the extracellular level of 5-HT *in vivo* in the prefrontal cortex, and inhibited the 5-HT transporter (Lord et al., 2014). In contrast, another study found reduced 5-HT level in the spinal cord in response to the inhibition of P2X7 in the rostral ventromedial medulla (Huang et al., 2014). Nevertheless, regulation of 5-HT release by *P2rx7* from nerve terminal subpopulations originating in raphe nuclei has remained unclear until now.

Therefore the objective of the present study was two-fold: at first, the optogenetic technique was used to selectively activate afferents originating from the MR locally and in one of its remote target areas, the HP to characterize the release of 5-HT originating from MRR subpopulation *in vivo* and *in vitro*, in response to a behaviorally relevant stimulus. To this end, adeno-associated virus expressing ChR2 was injected to MRR and photostimulation was used to release 5-HT *in vitro* and *in vivo*. The release of [^3^H]5-HT evoked by light stimulation from acute brain slices was compared with the effect of focal ES and chemical depolarization elicited by veratridine, respectively. Second, the modulation of electrical and OS induced [^3^H]5-HT release by *P2rx7*s was examined using gene deficient mice and specific antagonist of *P2rx7*s.

## Materials and Methods

### Mice

This study used drug and test naïve, 9–17-week old, male WT, and P2X7 knockout mice (KO). Breeding pairs of *P2rx7* homozygote knockout mice (C57BL/6J based; RRID:IMSR_JAX:000664) were originally supplied by Christopher Gabel (Pfizer Inc., Groton, CT, United States). The animals contained the DNA construct previously shown to produce genetic deletion of *P2rx7* (Solle et al., 2001). Homozygous knockout mice (KO) and WT (C57BL/6) littermates were bred in the local animal house (IEM HAS, SPF Unit). The genotype of the mice was tested by PCR analysis as previously described (Solle et al., 2001). Mice were housed under standard laboratory conditions with food and water available *ad libitum*, in a 12-h light–dark cycle at a temperature of 21–23°C.

All manipulations of the animals were approved by the local Animal Care Committee of the Institute of Experimental Medicine (Budapest, Hungary, ref. No. 22.1/361/3/2011) and performed according to the European Communities Council Directive recommendations for the care and use of laboratory animals (2010/63/EU).

### Virus Injection

**FIGURE 1:**
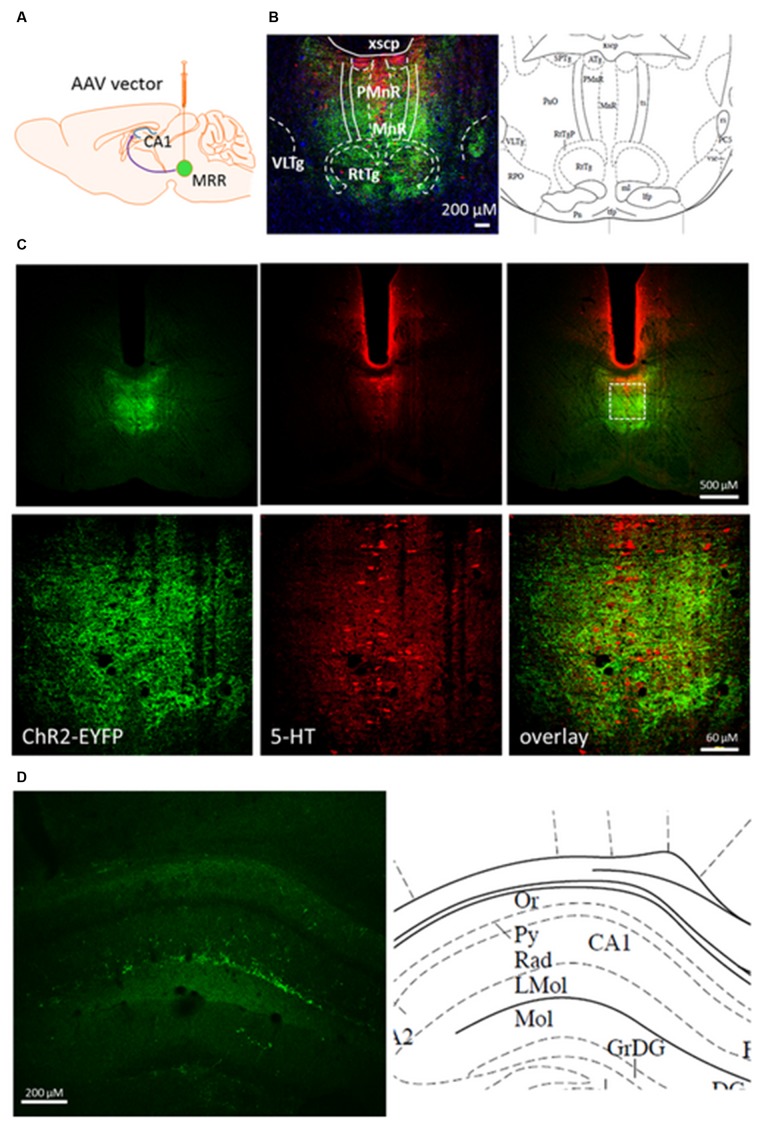
Coronal section from pontine area and dorsal hippocampus of rAAV-injected mouse brain 8 weeks after. **(A)** Mice were injected with viral vector in the MRR. **(B)** Immunohistochemically enhanced EYFP marker protein (green) and 5-HT containing cells (red) in the raphe region (–4.60 mm from Bregma) by confocal microscopy. Cell nuclei (blue) was stained by Hoechst 33258. **(C)** In an injected mouse, 5-HT (red) is expressed in many EYFP-labeled neurons (green) along the midline. Under, show the zoom-in view of the dashed rectangular area. **(D)** EYFP containing neuronal projections (green) originate from the MR in the str. radiatum of dorsal part of CA1, –1.82 mm from Bregma. Corresponding regions and abbreviations of typical nuclei in the given area shown from The Mouse Brain by Paxinos (Paxinos and Franklin, 2012). Lmol, lacunosum moleculare layer of the hippocampus; MnR, median raphe nucleus; Or, oriens layer of the hippocampus; PMnR, paramedian raphe nucleus; Py, pyramidal cell layer of the hippocampus; Rad, stratum radiatum of the hippocampus; RtTg, reticulotegmental nucleus of the pons; VLTg, ventrolateral tegmental area; xscp, decussation of the superior cerebellar peduncle. **(A)** was created by modifying images purchased in the PPT Drawing Toolkits-BIOLOGY Bundle from Motifolio, Inc. (http://www.motifolio.com/neuroscience.html).

Under intraperitoneal ketamine (160 mg/kg; Produlab Pharma b.v., Netherlands), and xylazine (6.7 mg/kg; Produlab Pharma b.v., Netherlands) anesthesia mice were held in a small animal stereotaxic apparatus by snout clamp (Kopf instruments, United States). 40 nl adeno-associated virus vector (AAV) encoding ChR2 (AAV2.5.hSyn.hChR2(H134R)eYFP.WPRE.hGH; 2.04e13 GC/ml; Addgene26973, Penn Vector Core, Philadelphia, PA) was injected from glass capillary (tip diameter 20–30 μm) connected to a MicroSyringe Pump Controller (World Precision Instruments) into the MRR (AP: 4.10 mm; L: 0.0 mm; DV: 4.60 mm, shown in **Figure 1A**) (Paxinos and Franklin, 2012). The capillary was left in place 5 min after the injections to permit diffusion of the virus and to minimize backflow of the virus after needle retraction. Immediately following injection the incision was closed and the scalp was sutured.

### Optogenetic Manipulation of Median Raphe Region

**FIGURE 2:**
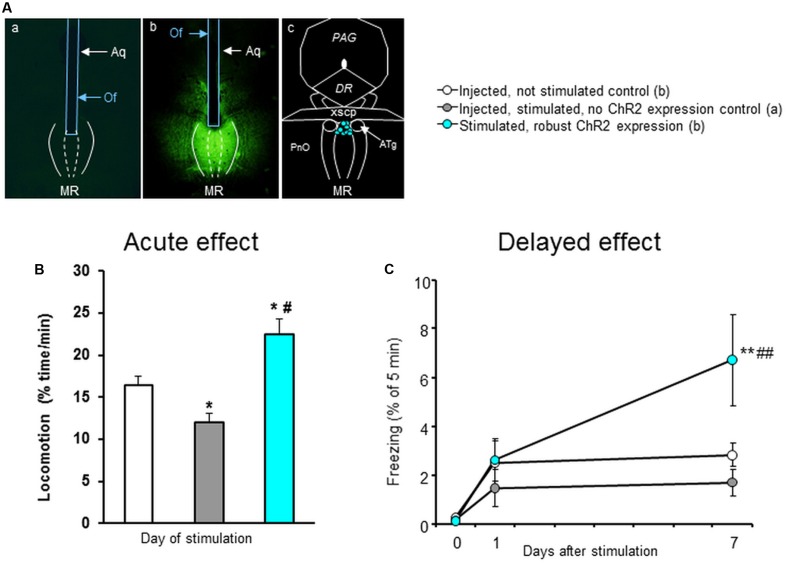
Optogenetic stimulation of MRR in a novel cage. **(A)** Representative photomicrographs showing the distribution of ChR2 in the MR (as visualized by GFP distribution) in mice where no ChR2 expression was present (a) or where it was robust (b) and the location of optrode tips in stimulated mice (c). **(B)** Locomotion counts, calculated as line crossings, for the whole duration of the experiment. **(C)** Freezing behavior during the 20 Hz stimulation (Day0) and 1 (Day1) and 7 days (Day7) later. *Aq*, aqueduct; *ATg*, anterior tegmental nucleus; *DR*, dorsal raphe; *MRR*, median raphe region; *Of*, optic fiber; *PAG*, periaqueductal gray; *xscp*, decussation of the superior cerebellar peduncle; Results were analyzed by one-way ANOVA **(B)**, and two-way repeated measure ANOVA **(C)**, followed by Newman–Keuls test, ^∗^ *p* < 0.05 or ^∗∗^*p* < 0.01 significant difference from injected, not stimulated control; #*p* < 0.05 or ##*p* < 0.01 significant difference from injected, stimulated, no ChR2 expression control.

Two weeks after injection of the AAV construct mice were implanted with optic fibers above the MRR (in an angle of 10°; AP:-4.80 mm; L: 0.0 mm; DV: 4.062) under ketamine-xylazine anesthesia (**Figure 2A**). Optic fibers for implantation and light stimulation were custom made from multimode optical fiber (AFS 105/125Y, NA: 0.22, low-OH, Thorlabs Corp.) and flanged zirconia ferrule (LMFL-172-FL-C35-OSK, Senko). Implants were secured by acrylic resin (Duracryl Plus; SpofaDental, Czech Republic). Behavioral experiments started after a 4–7 days recovery period. Laser beams were generated by low noise diode-pumped solid-state lasers [IkeCool Corp., wavelength: ChR2: 473 nm (blue)], then collimated and guided to the implanted optrode by fiber-optic patch cords (FT900SM, Thorlabs Corp.). Net energy output was measured by laser power meter (Coherent, LaserCheck) before and after the experiments, and data were considered only when optrodes transferred min. 10 mW power at continuous light emission. Mice were stimulated continuously at 20 Hz for 5 min (Day 0; *n* = 9) in Plexiglas stimulation cages measuring 30 × 30 × 30 cm. Controls were injected, not stimulated (*n* = 14) and light-stimulated mice, which showed no ChR2 expression in the MRR (*n* = 9). Behavior was recorded by a video camera, and later analyzed by experimenters blind to treatment conditions by a computer-based event recorder (H77, Budapest, Hungary). Locomotion was analyzed by counting line crossings (with all four legs) of a 3 × 3 grid that divided the cage into nine 10 × 10 cm squares. Lines were drawn on the video screen, that is, these were not visible to the mice during testing. Freezing is defined as complete immobility, except breathing. Freezing was analyzed during the 20 Hz stimulation (Day0), and 1 (Day1) and 7 days (Day7) later, when mice were replaced back to the stimulation cage. After behavioral studies, animals were perfused and histological analysis was performed. Mice with robust ChR2 expression and with correctly placed optrodes were included into the study.

### [^3^H]5-HT and [^3^H]Glu Release Experiments

Virus infected mice were used 8 weeks after injection; in other experiments, naïve mice of the same age were used. Briefly, mice were decapitated and acute 300–400-μm-thick coronal brain slices containing the MRR or HP was prepared using a vibratom in ice-cold Krebs’ solution (NaCl 113 mM, KCl 4.7 mM, KH_2_PO_4_ 1.2 mM, MgSO_4_ 1.2 mM, CaCl_2_ 2.5 mM, NaHCO_3_ 25 mM, glucose 11.5 mM, ascorbic acid 0.3 mM, Na_2_EDTA 0.03 mM, pH 7.4). Brain slices were then incubated in 1 ml of Krebs’ solution containing [^3^H]5-HT or [^3^H]Glu (5–5 μCi/ml) for 60 min at 37°C. Then tissues were transferred into low volume superfusion chambers and superfused with preheated ([^3^H]5-HT:37°C/[^3^H]Glu: 23°C) Krebs’ solution saturated with 95% O_2_ and 5% CO_2_ with 1 ml/min for 60 min. [^3^H]Glu release experiments were performed at lower temperature in order to minimize the spontaneous firing of CA1 and CA3 pyramidal cells and the metabolic efflux of glutamate (Sperlagh et al., 2002).

**FIGURE 3:**
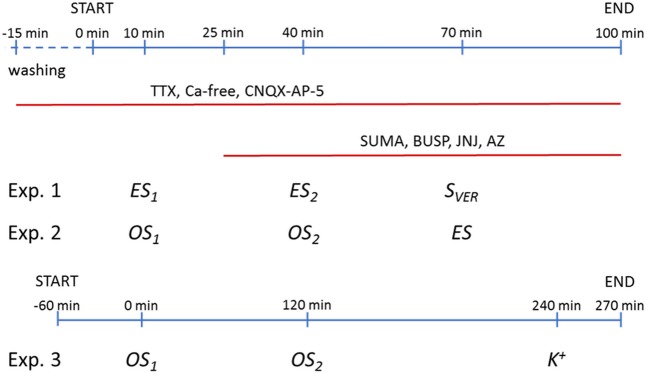
Overview of the experimental protocol of *in vitro* and *in vivo* neurotransmitter release experiments. Ca^2+^-free Krebs’ solution (Ca-free) was perfused from 60 min before the start the sample collection in radiolabelled neurotransmitter release experiments, while TTX and CNQX-AP-5 was administered 15 min before first collected sample and until the end. Sumatriptan (SUMA), buspirone (BUSP), JNJ-47965567 (JNJ) and AZ-10606120 (AZ) were added the perfusion solution 15 min before second light stimuli and until the end. Exp, experiment; *OS*, optical stimulation; *ES*, electrical stimulation; *S*_VER_, chemical stimulation (veratridine); *K^+^*, chemical stimulation (KCl).

After termination of the 1 h washing period, 1 or 3 min perfusate samples were collected and assayed for tritium. In Experiment 1 (**Figure 3**), at 10 and 40 min after the start of the sample collection period tritium overflow was evoked by two identical periods of electrical stimulation (*ES*_1-2_: 10–100 Hz, 3 V, 5–10 ms pulse width) by bipolar electrode placed to the slice surface, followed by a 3 min perfusion with the Na^+^ channel activator veratridine at 70 min (20 μM, *S*_VER_). In Experiment 2 (**Figure 3**), two optical stimulations (*OS*_1-2_: 10–100 Hz, 473 nm, 200 mW, 10 ms pulse width) were delivered by a Grass S 88 stimulator-controlled DPSS laser (IkeCool) coupled to an optic fiber with 105 micron core diameter submerged to the tissue chamber at the same time points, followed by one electrical stimulation (*ES*: 10–100 Hz, 3 V, 10 ms) at 70 min. When the frequency-dependence of the tritium efflux by different stimulations was examined, only the frequency of the stimulation was changed, with the exception of 100 Hz, when impulse duration was set at 5 ms, as 100 Hz with 10 ms would result in a continuous stimulation. Drugs and calcium-free Krebs’ solution were introduced 15/60 min before the first electrical/optical stimulation. The radioactivity released from the preparations was measured using a Wallac 1409 liquid scintillation counter (PerkinElmer, United States). Tritium efflux was expressed in Becquerel per gram (Bq/g) and as a percentage of the amount of radioactivity in the slices at the beginning of the respective collection period (fractional release, FR%). Optical or electrical stimulation-induced tritium efflux (*FRS*_x_) were expressed by calculating the net release in response to optical or electrical stimulations by the area-under-the-curve method.

### *In Vivo* Microdialysis

Eight weeks after injection of the AAV construct mice were anesthetized with 20% urethane (Reanal; Budapest, Hungary) and microdialysis probe (EICOM CX-I Brain Probe (membrane: artificial cellulose, molecular weight (MW) cut off: 50,000 Da, OD: 0.22 mm, length: 2 mm) were inserted into the MRR (an angle of 10° dorsal, AP:-4.80; L: 0.0 mm; DV: 5.50). An optic fiber (AFS 105/125Y, NA: 0.22, low-OH, Thorlabs Corp) was inserted through a guide cannula ending at the top of the membrane. After 2 h equilibration period, we collected 12 30 min samples, the first three samples served as baseline with a perfusion rate of 2 μl/min. Stimulation was delivered in the fourth (20 Hz) and eighth (50 Hz theta burst frequency: bursts with intraburst frequency of 50 Hz and interburst interval of 200 ms = 5 Hz) sample starting after 5 min after the beginning of sample collection and lasting 5 min (Experiment 3, **Figure 3**). The optic fiber transferred 10–20 mW net energy at continuous light emission. In the last sample 100 mM KCl was applied for 5 min.

### HPLC Analysis of ATP and Other Neurotransmitters *in Vivo* and *in Vitro* Samples

Neurotransmitters serotonin (5-HT), Glu and GABA in dialysates from MRR and endogenously released ATP in tissue over-flow fluid from HP slices were determined by using HPLC method. The extraction solution (PCA) was 0.1 M perchloric acid that contained theophylline (as an internal standard) at 10 μM concentration.

Initial volume of dialysis samples was measured and then diluted with an equal volume of ice cold PCA then supplemented with mobile phase “A” to 300 μL. The sample was centrifuged at 3510 *g* for 10 min at 0–4°C and 240 μL was injected onto the enrichment column. The remainder (60 μL) of the microdialysis sample was diluted with distilled water and the pH was adjusted to 10.5 with 2.7 M Na_2_CO_3_. The samples were reacted with (20 μL) 20 mM dansyl chloride for 15 min at 70°C temperature than the reaction was stopped by 10 μL formic acid. To determine Glu and GABA content, the volume of 350 μL of the reaction mixture was injected onto the enrichment column.

*In vitro* slice superfusate initial volume was measured then 50 μL ice cold PCA solution was added to the samples centrifuged as described above and 500 μL was used to determine the content of released ATP.

The levels of 5-HT and ATP were determined by online column switching separation using Discovery HS C18 50 × 2-mm and 150 × 2-mm columns. The flow rate of the mobile phases [“A” 10 mM potassium phosphate, 0.25 mM EDTA “B” with 0.45 mM octane sulphonyl acid sodium salt, 8% acetonitrile (v/v), 2% methanol (v/v), pH 5.2] was 350 or 450 μl/min, respectively in a step gradient application. The enrichment and stripping flow rate of buffer [10 mM potassium phosphate, pH 5.2] was during 4 min in micro dialysis and 8 min in superfusate samples. The total runtime was 55 min. The HPLC system used was a Shimadzu LC-20 AD Analytical & Measuring Instruments System, with an Agilent 1100 Series Variable Wavelength Detector set at 253 nm and an electrochemical (EC) amperometric detector BAS 400, Bioanalytical System set at 730 mV potential.

The levels of dansylated amino acids (Glu and GABA) were separated by the above column system. The flow rate of mobile phases [“A” 10 mM ammonium formate, 16.8% acetonitrile (v/v), methanol 4.8% (v/v), “B” 10 mM ammonium formate, 70% acetonitrile (v/v), methanol 20% (v/v), pH 3] was 400 μl/min in a linear gradient mode. The enrichment and stripping flow rate of the buffer [10 mM ammonium formate, 1.9% acetonitrile (v/v), 1.1% methanol (v/v)] was 300 μL/min during 4 min and the total runtime was 55 min. The used analytical system was the above, Shimadzu LC-20 System, with Gilson Model 121 Fluorimeter set at 340 nm excitation and 450 nm emission wavelength.

The recovery of the implanted microdialysis probes was evaluated at the end of experiment. The *in vitro* extraction efficiency for 5-HT, Glu, and GABA were estimated to be 21.1 ± 4.8%, 17.1 ± 2.8%, and 21.9 ± 3.4% respectively. The concentrations of 5-HT, Glu, and GABA were expressed in absolute amount (nmol/ml or pmol/ml) or as percentage (mean ± SEM) of baseline concentrations in order to monitor changes from basal levels after optical and chemical stimulation after virus injection of mice.

### Histological Verification

After behavioral and microdialysis experiments brains were taken out from deeply anesthetized mice after transcardial perfusion with 0.1M PBS for 1 min, followed by 4% paraformaldehyde in PBS for 10 min, and post-fixed for 24 h in fixative at +4°C. Afterward brains were cryo-protected by 20% glucose-PBS solution. Frozen coronal sections (40 μm thick) were cut by sliding microtome. Every third section was permeabilized by 0.5% Triton X-100 (Calbiochem) for 30 min and blocked with 2% bovine serum albumin (Sigma–Aldrich) for 30 min, both dissolved in PBS. Primary antibodies were diluted in PBS (Rabbit-anti-Serotonin, 1:10000, ImmunoStar, CatNo: 20080, **RRID**: AB_572263; Chicken anti-GFP, 1:2000, Life Technologies, CatNo: A10262, **RRID**: AB_2534023) and sections were incubated for 2 days at +4°C. Sections were then thoroughly washed in PBS and incubated in secondary antibody solution overnight (Cy3-conjugated Donkey-anti-Rabbit, 1:500, Jackson Immunoresearch, CodeNo: 711-165-152, **RRID**: AB_2307443; Alexa488-conjugated Goat-anti-Chicken, 1:1000, Life Technologies, CatNo: A-11039, **RRID**: AB_2534096; diluted in PBS). After multiple PBS washes, sections were mounted on slides and cover-slipped with Mowiol (Calbiochem) containing 10 μg/ml bisbenzimide (Hoechst 33258, Sigma). After release experiments the slides were fixed by 4% paraformaldehyde and mounted on slides without further processing for detection of the fluorescent signal of the virus construct. Sections were evaluated and images were taken with a Nikon C2 confocal microscope. The position of the optic fiber, microdialysis probe and the virus infected volume were determined on micrographs by using stereotaxic atlas images on the series of images of the MRR (Paxinos and Franklin, 2012).

### Drugs

[^3^H]5-HT (5-hydroxytryptamine[^3^H(G)] (serotonin), spec. activity 113.2 Ci/mmol) and [^3^H]Glu (Glutamic acid, L-[2,3,4-3H], spec. activity 60 Ci/mmol) were obtained from American Radiolabelled Chemicals Inc. Tetrodotoxin (TTX, from Alomone labs), CNQX (disodium salt; Tocris), DL-AP-5 (Tocris), sumatriptan (Sigma), buspirone (Sigma) and AZ-10606120 (Tocris) were dissolved in distilled water, veratridine (Tocris) was dissolved in DMSO. JNJ-47965567 (Tocris) was dissolved in 30% sulfobutylether beta-cyclodextrin (SBE, from Ligand Pharmaceuticals Inc.). The maximum concentrations of vehicles used had no significant effect on the [^3^H]5-HT/[^3^H]Glu release. All solutions were freshly prepared on the day of use.

### Statistics

All data represent mean ± SEM. *P*-values of less than 0.05 were considered statistically significant. STATISTICA software (**RRID**: SCR_014213) was used for all statistical analyses. Locomotion counts were analyzed by one way ANOVA with “treatment” and “stimulation” variables. Pairwise comparisons were made by the Newman–Keuls *post hoc* test. In freezing behavior the groups were compared using two-way repeated measures ANOVA with “treatment” and “time” variables followed by Newman–Keuls *post hoc* test. In release and other experiments the normality of data distribution were tested by the Shapiro–Wilk normality test. When the data were normally distributed or log-transformed, drug treatment or stimulation effects were tested by one-way ANOVA followed by Dunnett or Scheffe *post hoc* test. If not, data were analyzed by Kruskal–Wallis test with Dunn’s *post hoc* test. HPLC data were analyzed by two-way ANOVA for factorial measures, between “treatment” and “stimulation” as variables. Tukey’s and Fischer LSD *post hoc* comparisons were used where appropriate.

## Results

### Expression of ChR2-EYFP in MR and HP Neurons in Mice

We used the optogenetic method to selectively stimulate the neurotransmitter release from MR neurons and nerve terminals *in vitro* and *in vivo*. To visualize the expression of the virus construct, at first we examined the distribution of ChR2-EYFP-expressing cells in MR (**Figures 1B,C**) and HP (**Figure 1D**) 8 weeks after rAAV injection. Confocal images of coronal section revealed the robust and circumscript expression of ChR2-EYFP in both 5-HT positive and negative cells of MRR, but not DR following injection of rAAV (**Figure 1C**). In the hippocampal region, the terminals fields at the border of *stratum radiatum* and *stratum lacunosum moleculare* displayed the most intensive ChR2-EYFP labeling (**Figure 1D**).

### Optogenetic Stimulation of MRR ChR2-Expressing Neurons Induces Changes in Locomotion and Freezing

Next, we examined the behavioral relevance of the optogenetic activation (20 Hz, 5 min, 473 nm) of MRR on locomotor activity and freezing in mice *in vivo* (**Figure 2**). We found that the locomotion of mice was affected by the light stimulation (*^∗∗^p* < 0.01). *Post hoc* analysis revealed that the light stimulation in control, no ChR2 expression mice (*n* = 9) reduced the locomotion as compared to no light controls (*^∗^p* < 0.05, *n* = 14) suggesting a possible thermal effect (Stujenske et al., 2015). However, optogenetic stimulation of MRR (*n* = 9) resulted in an enhanced locomotion in comparison to both (non-stimulated and no ChR2 expression) control groups (*^∗∗^p* < 0.01, and *^∗∗^p* < 0.01, respectively) (**Figure 2B**). 20 Hz stimulation had no effect either on stimulation day (Day0) nor next day (Day1), but increased freezing behavior on Day 7 (stimulation: *^∗^p* < 0.05; time: *^∗∗^p* < 0.01; interaction: *^∗^p* < 0.05) (**Figure 2C**). Therefore optogenetic activation of MRR ChR2-expressing neurons induced a long-term change in the behavioral responsiveness of the animals.

### Electrical and Optical Stimulation Induced [^3^H]5-HT Release from Median Raphe Region

**FIGURE 4:**
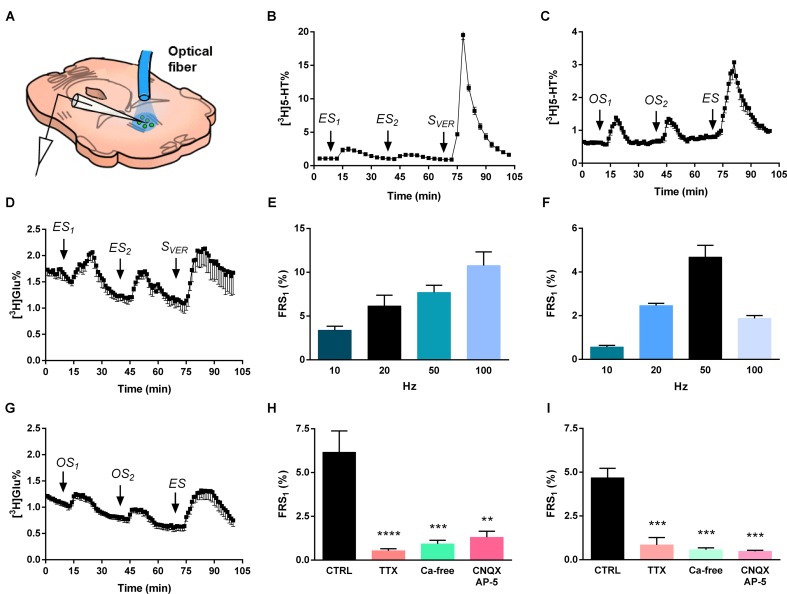
Electrical and optical stimulation induces release of [^3^H]5-HT and [^3^H]Glu from the MRR. **(A)** [^3^H] neurotransmitter release was measured from superfused coronal slices containing the MRR of virus-injected mice in response to different stimulations. The schematic figure illustrates the position of optical fiber and bipolar electrode. **(B,C)** [^3^H]5-HT release in response to electrical (20 Hz) **(B)** and optogenetic (50 Hz) **(C)** stimulation. *ES* is an electrical, *S*_VER_ is a chemical (veratridine, 20 μM) and *OS* is a light stimulation, *n* = 4–5 mice. **(E,F)** Frequency-dependence of [^3^H]5-HT release from MRR, *n* = 4–5 mice on each frequency. We used different frequencies (10, 20, 50, 100 Hz) with unchanged other stimulation parameters. **(H,I)** Effects of TTX, CNQX-DL-AP-5 and Ca^2+^-free conditions on the electrical (20 Hz) **(H)** and optical (50 Hz) **(I)** stimulation-evoked release of [^3^H]5-HT from MRR. Ca^2+^-free Krebs’ solution was perfused from 60 min before the start the sample collection, while TTX and CNQX-AP-5 was administered 15 min before first collected sample and until the end, *n* = 4 mice per group. **(D,G)** 50 Hz electrical stimulation **(D)** and 50 Hz optical stimulation **(G)** induces release of [^3^H]Glu from MRR, *n* = 3–6 mice. The tritium content in the perfusate samples **(B–D,G)** was expressed as fractional release (FR%) and is shown as a function of time. Curve shows the mean ± SEM of the identical experiments. Arrows indicate the time of different type of light, electric and chemical stimulations. **(E,F,H,I)** The results are expressed as the net release of tritium evoked by the first electrical or optical stimulation period (*FRS*_1_, %). **(H,I)** Results were analyzed by one-way ANOVA, followed by Dunnett test, ^∗∗∗∗^*p* < 0.0001, ^∗∗∗^*p* < 0.001, ^∗∗^*p* < 0.01. Asterisks indicate significant differences from control. **(A)** image is a modification of the original one from the article of Dugue et al. (2014).

Then, we examined, whether a similar optogenetic activation *in vitro* results in a neurochemically detectable transmitter overflow, and to compare its effect with electrical and chemical depolarization. The experimental protocols used in these experiments are shown on **Figure 3**. The placement of optical fiber and stimulating electrode is shown on **Figure 4A**, the time course of [^3^H]5-HT release measured from MRR slices induced by electrical, optical and chemical depolarization stimuli of the mouse is shown in **Figures 4B,C**.

In Experiment 1 (**Figure 4B**) two identical periods of electrical stimulation (*ES*_1-2_) was followed by a 3 min perfusion with the Na^+^ channel activator veratridine (20 μM, *S*_VER_). ES (3 V, 5 ms, 20 Hz, 10800 pulses) elicited a transient elevation of [^3^H]5-HT outflow (*FRS*_1_: 6.16 ± 1.22 FR%) (**Figure 4B**). A subsequent, identical stimulation caused a similar, but smaller increase in the [^3^H]5-HT outflow, resulting in a *FRS*_2_/*FRS*_1_ ratio of 0.49 ± 0.06. Chemical depolarization by veratridine at the end of the experiment elicited a robust increase in the released radioactivity (*FRS*_VER_: 50.5 ± 1.72 FR%), indicating that the releasable pool of [^3^H]5-HT was not exhausted by the previous stimulations. Further, we have examined the effects of ES of different frequencies (10, 20, 50, and 100 Hz) on [^3^H]5-HT release from MRR (**Figure 4E**), without changing the voltage and the pulse width. The efflux of tritium released by ES was frequency- (**Figure 4E**) and [Ca^2+^]_o_- dependent (*^∗∗∗^p* = 0.00022), and sensitive to the inhibition of the voltage gated Na^+^ channel blocker TTX (1 μM) (*^∗∗∗∗^p* = 0.00003) (**Figure 4H**). Inhibition of NMDA and non-NMDA receptors by co-administration of DL-AP-5 (50 μM) and CNQX (10 μM) also inhibited the majority of tritium efflux evoked by ES (*^∗∗^p* = 0.00131) (**Figure 4H**).

Next, we examined how slices respond to selective activation of MRR neurons expressing the ChR2-EYFP construct. Because preliminary experiments showed that photostimulation does not result in a detectable [^3^H]5-HT efflux at an earlier time point (see Supplementary Figure 1) experiments were performed 8 weeks after virus delivery. In Experiment 2 two optical stimulations (*OS*_1-2_: 50 Hz, 473 nm, 10800 pulses) were delivered, followed by one electrical stimulation (*ES*: 50 Hz, 3 V, 10800 pulses) at 70 min (**Figure 4C**). OS elicited reproducible [^3^H]5-HT efflux from MRR slices (*FRS*_1_: 4.68 ± 0.54 FR%, *FRS*_2_/*FRS*_1_: 1.101 ± 0.29), which was substantially less, than the tritium release evoked by the subsequent ES with identical frequency and impulse duration (**Figure 4C**). OS of MRR derived from control, naïve mice with identical parameters did not elicit any increase in tritium efflux, excluding any non-specific effects of OS (see Supplementary Figure 2). We found that OS induced an increase in [^3^H]5-HT outflow at all frequencies (**Figure 4F**), however, the tritium efflux was frequency-dependent only within the interval of 10–50 Hz. TTX (1 μM) almost completely abolished the optically evoked tritium release in this brain area (*FRS*_1_: 0.85 ± 0.42 FR%) (^∗∗∗^*p* = 0.00063) at 50 Hz (**Figure 4I**). These data imply that 5-HT released in response to light stimulation is associated with propagating sodium channel activity in MRR. Likewise, [Ca^2+^]_o_ - free conditions (*FRS*_1_: 0.57 ± 0.10 FR%) (^∗∗∗^*p* = 0.00063), and blockade of the excitatory neurotransmission by DL-AP-5 (50 μM), plus CNQX (10 μM) induced a decrease in the release of [^3^H]5-HT from MRR (*FRS*_1_: 0.48 ± 0.07 FR%) (^∗∗∗^*p* = 0.00033) (**Figure 4I**). These findings indicated that tritium efflux originating from the ChR2 expressing neurons is partly mediated by endogenous Glu receptor activation within the MRR.

To further explore this possibility, we preloaded slices with [^3^H]Glu and examined, whether light stimulation with identical parameters (50 Hz, 473 nm, 10 ms, 10800 pulses) induces tritium outflow from the MRR. This was exactly the case as OS elicited a reproducible increase in [^3^H]Glu outflow (*FRS*_1_: 1.91 ± 0.33 FR%), which was comparable, but less than the effect of the ES (see **Figures 4D,G**, and **Table 1** for an overview).

**Table 1:** Comparison of uptake and release of [^3^H]5-HT and [^3^H]Glu in mice median raphe and hippocampus.

Experimental group	[^3^H] uptake ( × 10^5^ Bq/g)	Resting release (%)	ES_1_ Evoked release ( × 10^5^ Bq/g)	S_V ER_ Evoked release ( × 10^5^ Bq/g)	Genotype
**Experiment 1**					
5-HT MR (*n* = 4)	5.814 ± 2.217^∗∗∗^	0.86 ± 0.04	0.288 ± 0.153	1.155 ± 0.459	WT
Glu MR (*n* = 3)	58.532 ± 13.238	0.96 ± 0.01	1.059 ± 0.692	1.579 ± 0.593	WT
5-HT HP (*n* = 4)	1.373 ± 0.182^$$$^	0.99 ± 0.09	0.045 ± 0.002	0.135 ± 0.019	WT

**Experimental group**	**[^3^H] uptake ( × 10^5^ Bq/g)**	**Resting release (%)**	**OS_1_ Evoked release ( × 10^5^ Bq/g)**	**ES Evoked release ( × 10^5^ Bq/g)**	**Genotype**

**Experiment 2**					
5-HT MR (*n* = 5)	16.670 ± 2.888^∗∗∗^	1.83 ± 0.08^###^	0.634 ± 0.083	1.242 ± 0.299^∗^	WT
Glu MR (*n* = 8)	51.425 ± 6.237	2.43 ± 0.18	0.622 ± 0.105	2.583 ± 0.334	WT
5-HT HP (*n* = 5)	11.761 ± 1.939^$$$^	4.38 ± 0.33^$$$^	0.247 ± 0.085^$^	0.526 ± 0.096^$$$^	WT
5-HT HP (*n* = 5)	13.617 ± 2.014^$$$^	4.58 ± 0.28^$$$^	0.172 ± 0.039^#,$^	0.264 ± 0.077^$$$^	KO


*At 10 and 40 min after the start of the sample collection period tritium overflow was evoked by two identical periods of electrical (50 Hz, Experiment 1) and optical stimulation (50 Hz, Experiment 2), followed by a 3 min perfusion with the Na^+^ channel activator veratridine at 70 min (20 μM, *S*_VER_, Experiment 1) or 50 Hz electrical stimulation (Experiment 2). Means ± SEM of *n* observation are presented. The number of experiments is given in parenthesis. 5-HT MR vs. Glu MR ^∗^*p* < 0.05, ^∗∗∗^*p* < 0.001; 5-HT MR vs. 5-HT HP ^#^*p* < 0.05, ^###^*p* < 0.001; 5-HT HP vs. Glu MR ^$^*p* < 0.05, ^$$$^*p* < 0.001, calculated by one-way ANOVA followed by the Tukey’s *post hoc* test.*

### Electrical and Optical Stimulation Evoked [^3^H]5-HT Release from the HP

**FIGURE 5:**
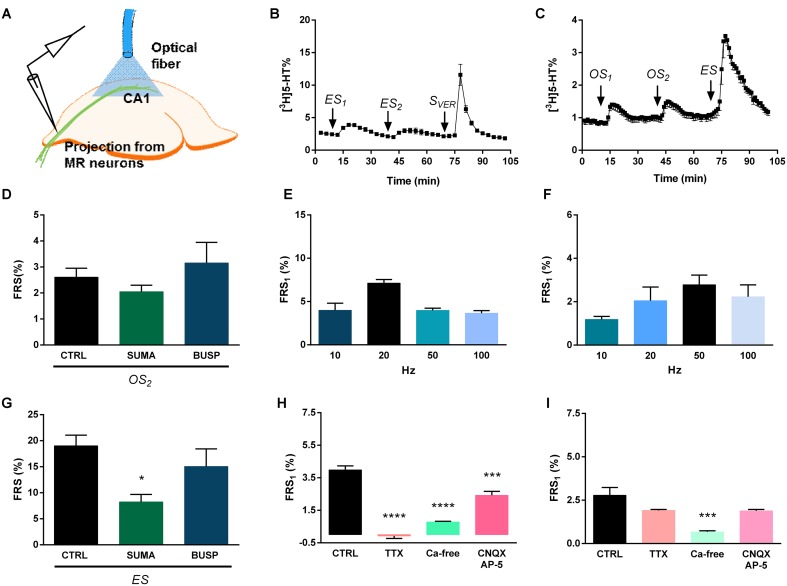
Electrical and optical stimulation results release of [^3^H]5-HT from superfused HP slices. **(A)** [^3^H] neurotransmitter release was measured from HP slices of virus-injected mice in response to different stimulations. The schematic figure illustrates the position of optical fiber. **(B,C)** [^3^H]5-HT release in response to electric (20 Hz) **(B)** and optogenetic (50 Hz) **(C)** stimulation. *ES* is an electrical stimulus, *S*_VER_ is a chemical (veratridine, 20 μM) stimulus, and *OS* is a light stimulation, *n* = 4–5 mice. **(E,F)** Frequency-dependence of [^3^H]5-HT release (10–100 Hz) from HP slices, n = 4-5 mice on each frequency. **(H,I)** Effects of TTX, CNQX-AP-5 and Ca^2+^-free conditions on the electrical (50 Hz) **(H)** and optical (50 Hz) **(I)** stimulation-evoked release of [^3^H]5-HT from HP. Ca^2+^-free Krebs’ solution was perfused from 60 min before the start the sample collection, while TTX and CNQX-DL-AP-5 was administered 15 min before first collected sample and until the end, *n* = 4 mice per group. **(D,G)** Sumatriptan, but not buspirone significantly decreased the electrically evoked release of [^3^H]5-HT in HP slices. [^3^H]5-HT release in response to optogenetic (50 Hz) **(D)** and electric (50 Hz) **(G)** stimulation, *n* = 4–5 mice per group. Sumatriptan and buspirone were added the perfusion solution 15 min before second light stimuli. *OS*_2_ is a light stimulation and *ES* is an electrical stimulus. The tritium content in the perfusate samples **(B,C)** was expressed as fractional release (FR%) and is shown as a function of time. Curve shows the mean ± SEM of the identical experiments. Arrows indicate the time of different type of light, electric and chemical stimulations. **(D–I)** The results are expressed as the net release of tritium evoked by the first electrical or optical stimulation period (*FRS*_1_, %). **(D,G–I)** Results were analyzed by one-way ANOVA, followed by Dunnett test, ^∗∗∗∗^*p* < 0.0001, ^∗∗∗^*p* < 0.001, ^∗^*p* < 0.05. Asterisks indicate significant differences from control. **(A)** Created by modifying images purchased in the PPT Drawing Toolkits-BIOLOGY Bundle from Motifolio, Inc. (http://www.motifolio.com/neuroscience.html).

The placement of optical fiber is shown on **Figure 5A**, the time course of [^3^H]5-HT release measured from HP slices induced by electrical, optical and chemical depolarization stimuli of the mouse is shown in **Figures 5B,C**.

Electrical stimulation (20 Hz, 3 V, 5 ms, 10800 pulses) elicited a transient, but largely reproducible elevation of [^3^H]5-HT outflow in the HP (*FRS*_1_: 7.13 ± 0.42 FR%; *FRS*_2_/*FRS*_1_: 0.74 ± 0.18) (**Figure 5B**). Veratridine applied at the end of the experiment released 17.78 ± 1.69 FR% (*n* = 4 mice) of the actual tritium content (**Figure 5B**).

When the electrically evoked [^3^H]5-HT release was examined from HP slices (**Figure 5E**), no clear frequency dependence was observed and the highest elevation in tritium efflux was observed at 20 Hz. TTX (1 μM) almost completely abolished the electrically evoked tritium release (0.08 ± 0.15 FR%) (^∗∗∗∗^*p* = 0.0000001) (**Figure 5H**). The evoked release of [^3^H]5-HT was also substantially inhibited by Ca^2+^-free conditions (^∗∗∗∗^*p* = 0.00001) (**Figure 5H**). DL-AP-5 and CNQX significantly decreased electrically evoked [^3^H]5-HT (^∗∗∗^*p* = 0.00075) (**Figure 5H**).

The pattern of [^3^H]5-HT release by two optical and subsequent ES in Experiment 2 was similar to that observed in the MRR. Light stimulation (50 Hz, 473 nm, 10 ms) induced a smaller, but still well detectable tritium efflux to both consecutive stimulations (*FRS*_1_: 2.78 ± 0.45 FR%, *FRS*_2_/*FRS*_1_: 0.99 ± 0.08), in a comparable but less amount that the effect of the ES (**Figure 5C**). In case of light-stimulation induced [^3^H]5-HT efflux, a steeper frequency-dependence was observed, which reached a plateau at 50 Hz (**Figure 5F**).

In contrast to tritium release evoked by ES, neither TTX (1 μM), nor DL-AP-5 (50 μM) and CNQX (10 μM) affected significantly [^3^H]5-HT efflux evoked by light stimulation (TTX: *FRS*_1_: 1.91 ± 0.06 FR%, *p* = 0.14936; DL-AP-5 and CNQX: *FRS*_1_: 1.78 ± 0.12 FR%, *p* = 0.13499) illustrating the exclusive, direct effect of this type of stimulation on 5-HT releasing axon varicosities, originating in the MRR (**Figure 5I**). This release was also exocytotic in nature, as Ca^2+^ -free Krebs’ solution inhibited light stimulation-induced [^3^H]5-HT efflux from the HP (Ca^2+^ free: *FRS*_1_: 0.67 ± 0.07 FR%, *^∗∗∗^p* = 0.00061) (**Figure 5I**).

### Modulation of [^3^H]5-HT Release from HP by 5-HT1_B/D_ Terminal Autoreceptors

It is widely established that cortical 5-HT-ergic nerve terminals are equipped with presynaptic 5-HT1_B/D_ terminal autoreceptors, the activation of which inhibits the release of 5-HT (Trillat et al., 1997; Malagie et al., 2001). However, the origin of the nerve terminals expressing 5-HT1_B/D_ receptors in the HP remained unidentified so far. Thus, we tested whether [^3^H]5-HT efflux from the HP evoked by electrical and light stimulation of ChR2 expressing terminals, are subject to modulation by 5-HT1_B/D_ receptors. The 5-HT1_B/D_ agonist sumatriptan (SUMA, 1 μM), perfused before the second optical stimulation (*OS*_2_) using the same protocol mentioned above, significantly decreased tritium released by *ES* (*^∗^p* = 0.01201) (**Figure 5G**), but did not affect [^3^H]5-HT efflux evoked by OS (**Figure 5D**). In contrast, buspirone (BUSP, 0.1 μM), the selective 5-HT_1A_ agonist was without effect either on the light or ES-induced tritium efflux (**Figures 5D,G**).

### Modulation of Electrical and Optical Stimulation Evoked [^3^H]5-HT Release from the HP by P2X7 Receptors

**FIGURE 6:**
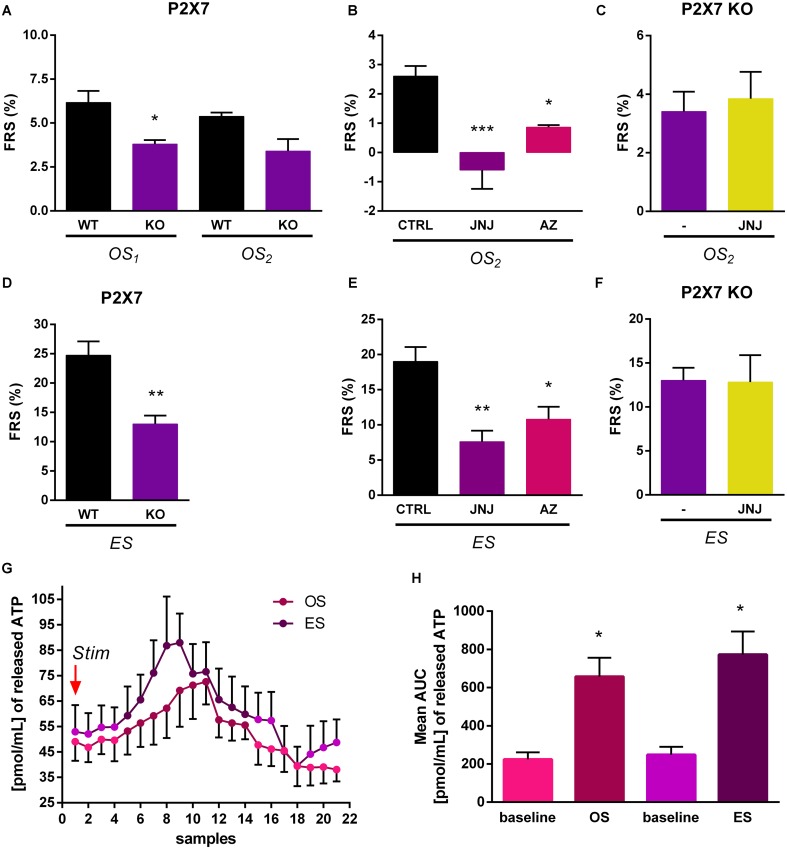
Optically and electrically evoked tritium efflux was lower in KO than in WT mice. **(A,D)** Optically (50 Hz) **(A)** and electrically (50 Hz) **(D)** induced tritium release in WT and KO mice (*n* = 5 mice per group). **(B,E)** JNJ-47965567(JNJ) and AZ-10606120 (AZ) significantly decreased the release of [^3^H]5-HT elicited by light (50 Hz) **(B)** and electrical (50 Hz) **(E)** stimulation from HP slices in WT mice (*n* = 4 mice per group). **(C,F)** Treatment with the P2X7 receptor antagonist, JNJ-47965567 did not decreased significantly the tritium efflux elicited by light, *OS*_2_ (50 Hz) **(C)** and electrical, *ES* (50 Hz) **(F)** stimulation from HP slices in KO mice (*n* = 4 mice per group). In every case, we used the protocol of Experiment 2 (see **Figure 3**) and JNJ-47965567 was added to the perfusion solution 15 min before second light stimuli. **(G,H)** Optical and electrical stimulations increased the endogenous ATP release in the HP of virus injected WT mice. We used the protocol of Experiment 2. Stim **(G)** indicates the first optical (OS_1_) and last, electrical stimulation (ES). The two types of stimulation applied at 50 Hz with same parameters, but not in the same time. *X*-axis shows the 1 min perfusate samples from the starting of stimulation. Graph in panel **(H)** represents the area under curve (AUC) values. Baseline is the mean ATP content expressed by AUC method before *OS* and *ES* stimulation. **(A–F)** *OS*_1_ and *OS*_2_ is a light stimulation and *ES* is an electrical stimulus. The results are expressed as the net release of tritium evoked by the optical or electrical stimulation period (*FRS*, %). **(G)** Data are expressed as pmol/mL of released ATP as means ± SEM (*n* = 6 per group). Results were analyzed by one-way ANOVA, followed by Scheffe **(A,C,D,F)** and Dunnett test **(B,E)**, and Kruskal–Wallis ANOVA with Dunn’s test **(H)**, ^∗^*p* < 0.05, ^∗∗^*p* < 0.01, ^∗∗∗^*p* < 0.001. Asterisks indicate significant differences from control.

Next we examined, whether [^3^H]5-HT efflux from the HP evoked by electrical and light stimulation of ChR2 expressing terminals is subject to modulation by endogenous activation of *P2rx7*s. To this end, we have compared tritium efflux evoked by the respective stimulations in WT and *P2rx7* deficient (KO) mice using the protocol of Experiment 2 (**Figure 3**). After loading the HP slices with [^3^H]5-HT, the tissue uptake of radioactivity was not significantly different in the slices from WT and KO mice (**Table 1**). Both electrical and optical stimulation elicited an increase in the efflux of [^3^H]5-HT in WT and KO mice, however, release was lower in the mice genetically deficient in *P2rx7*s (**Figures 6A,D**). Next, we tested the effect of two *P2rx7*s antagonists: JNJ-47965567 (100 nM), the potent and selective *P2rx7* antagonist (Bhattacharya et al., 2013) and AZ-10606120, the negative allosteric modulator of *P2rx7*s (100 nM) (Michel et al., 2008) on the release of [^3^H]5-HT evoked by light and electrical stimulation from HP slices of WT mice. We again used protocol of Experiment 2 (**Figure 3**). The OS-evoked [^3^H]5-HT efflux was almost completely inhibited by JNJ-47965567 (*^∗∗∗^p* = 0.00030) (**Figure 6B**) and the *P2rx7* antagonist also significantly decreased the release of [^3^H]5-HT elicited by electrical stimulation (**Figure 6E**). AZ-10606120 (100 nM) applied in an identical manner, also attenuated optical and electrical stimulation induced 5-HT efflux (*OS*_2_: *^∗^p* = 0.02027, ES: *^∗^p* = 0.01983) (**Figures 6B,E**). These data showed that the release of 5-HT by optical and electrical stimulation is subject to modulation by endogenous activation of hippocampal *P2rx7*s. We also tested the effect of JNJ-47965567 on the release of [^3^H]5-HT evoked by light and electrical stimulation from HP slices of P2X7 KO mice. JNJ-47965567 did not elicit any decrease in tritium efflux compared to the control mice (untreated with P2X7 antagonist) (see **Figures 6C,F**). To demonstrate that endogenous ATP is released under identical conditions, we have analyzed the ATP content of the samples by HPLC (**Figure 6G**). Optical and electrical stimulation elevated ATP level in the samples collected after the stimulation showing nucleotide release in response to stimulation of MRR terminals (**Figure 6H**).

### Effects of *in Vivo* Light and K^+^ Stimulation on 5-HT, Glutamate and GABA Levels in the MRR in a Microdialysis Study

**FIGURE 7:**
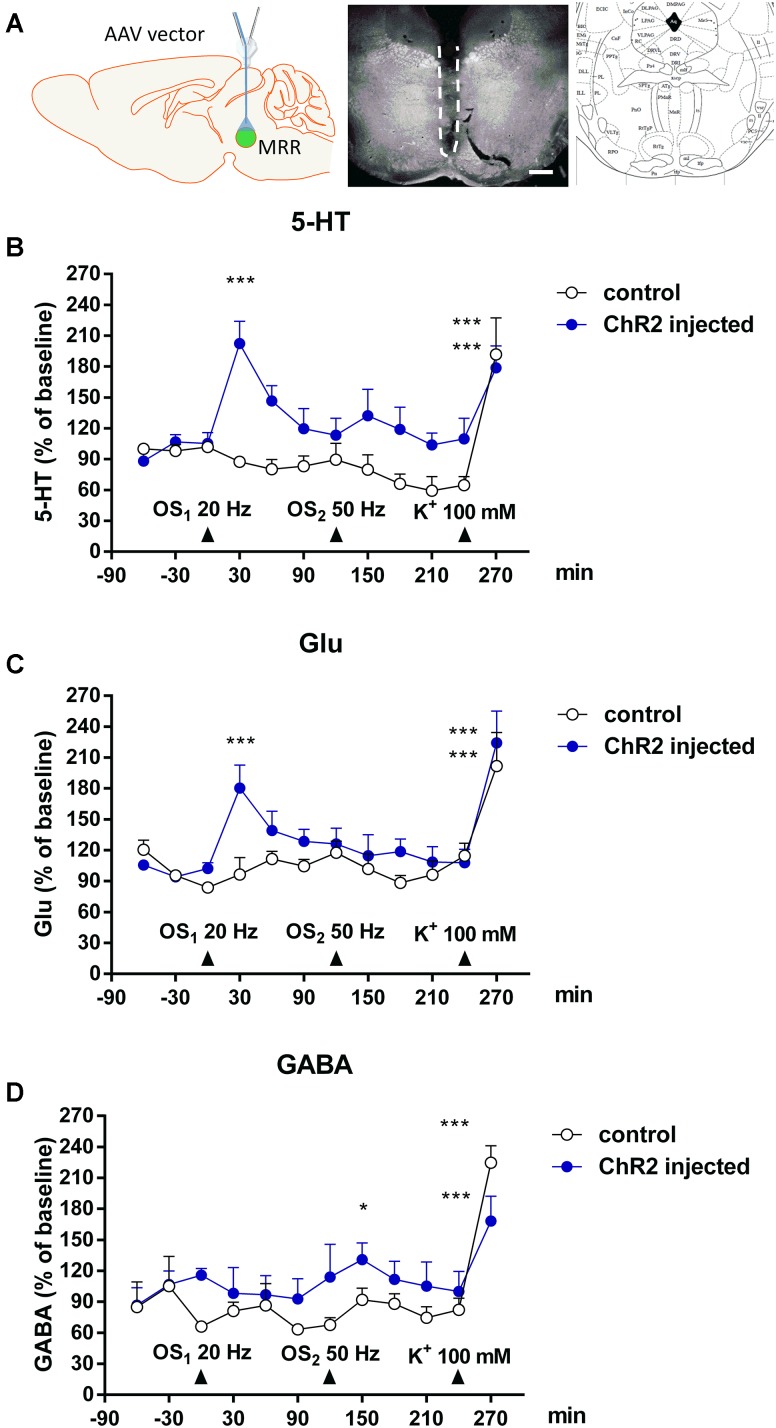
*In vivo* light and K^+^ stimulation increased 5-HT, Glu and GABA levels in MMR. **(A)** Left: The location of microdialysis probe and optic fiber in stimulated mice. Center: Dark field photomicrograph shows the injury (indicated by dashed line) caused by microdialysis probe in the dorsal (DR) and medial raphe nucleus (MR). Right: Insert from the Paxinos mouse brain atlas –4.60 mm from the Bregma shows the appropriate area. Scale bars: 500 μm. **(B,C)** Optical stimulations (*OS*_1_: 20 Hz 5 min) increased the 5-HT and Glu level in the MRR in virus injected mice. **(D)** Second optical stimulation (*OS*_2_: 50 Hz, 5 min) increased the GABA level in the MRR. **(B–D)** Arrows indicate the time of different type of light and chemical stimulations. Data are expressed as % of baseline as means ± SEM (*n* = 5 or 10 per group). Results were analyzed by two-way factorial ANOVA, followed by Dunnett / Fischer LSD (GABA) *post hoc* test ^∗^*p* < 0.05, ^∗∗∗^*p* < 0.001. Asterisks indicate significant differences from baseline. **(A)** Created by modifying images purchased in the PPT Drawing Toolkits-BIOLOGY Bundle from Motifolio, Inc. (http://www.motifolio.com/neuroscience.html).

Finally we assessed, whether local OS leads to the elevation of endogenous neurotransmitters in the MRR, measured by HPLC (**Figure 7**). After 2-h stabilization period, the basal levels of 5-HT, Glu and GABA reached a steady state in the MRR of all mice. The basal efflux of 5-HT, Glu and GABA were 1.66 ± 0.17 pmol/ml, 3.41 ± 0.43 nmol/ml and 0.46 ± 0.11 nmol/ml, respectively (*n* = 5 mice per group). In these experiments (Experiment 3, **Figure 3**) two consecutive 5-min light stimulations were applied with 20 and 50 Hz frequency, which was followed by a 5 min period of K^+^ depolarization (100 mM). Control mice did not contain the rAVV construct, but were optically stimulated.

Optical stimulation (20 Hz) significantly increased the extracellular levels of 5-HT (*F*_1,174_ = 20.2825, *^∗∗∗^p* = 0.00001) (**Figure 7B**) and Glu (*F*_1,174_ = 9.279, *^∗∗^p* = 0.0027) (**Figure 7C**) but not GABA (*F*_1,174_ = 0.0419, *p* = 0.8380) (**Figure 7D**) in MRR of virus infected mice. In absolute amount, the elevation of 5-HT and Glu levels reached 3.98 ± 0.66 pmol/ml (*F*_3,174_ = 4.0833, *^∗∗^p* = 0.0079, 213.7% of basal), and 4.76 ± 0.51 nmol/ml (*F*_3,174_ = 2.7149, *^∗^p* = 0.0465, 180.2% of basal), respectively. In control mice, identical light stimulation did not alter the basal extracellular 5-HT, Glu and GABA levels in MR (**Figures 7B–D**).

The next photostimulation was conducted with 50 Hz theta burst frequency (bursts with intraburst frequency of 50 Hz and interburst interval of 200 ms = 5 Hz). Using this protocol, we did not observe significant increase in Glu and 5-HT levels in the MRR (**Figures 7B,C**). In contrast, the GABA levels in MRR of virus infected mice increased to 1.40 ± 0.2 nmol/ml, (136.7% percent of basal values, *^∗^p* = 0.0205) (**Figure 7D**). Once again, no change was detected in the level of either transmitters in response to an identical OS in control mice (**Figures 7B–D**).

K^+^-depolarization increased the extracellular levels of 5-HT (*F*_3,174_ = 15.5803, *^∗∗∗^p* = 0.000001), Glu (*F*_3,174_ = 22.4020, *^∗∗∗^p* = 0.000001), and GABA (*F*_3,174_ = 17.8765, *^∗∗∗^p* = 0.000001) in the MRR of both virus injected and control mice, showing the viability and responsiveness of brain area at the end of the experiment (**Figures 7B–D**).

## Discussion

In this study we have used for the first time optogenetic stimulation to characterize neurotransmitter release in response to selective stimulation of neurons originating in the MRR. Whilst ES or chemical depolarization conventionally used to detect transmitter release excites synchronously all cell bodies, axons and nerve terminals in the stimulation field, the optogenetic technique allows the separate activation of a selected neuronal pathway (Melchior et al., 2015).

20 Hz OS caused an increase in the locomotor activity of the animals and elicited freezing behavior 7 days after the stimulation, indicating that this kind of stimulation is behaviorally relevant. Previous studies showed that ascending serotonergic projections play an essential role in such motor acts as walking, moving the head about, or changing posture or in general, in enhanced locomotion (Peck and Vanderwolf, 1991). Our recent studies shows that MRR stimulation alone generates remote, but not recent fear memory traces in addition to the known indirect control of fear circuitry, by influencing the encoding and retrieval of fear memories by amygdala, HP and prefrontal cortex (Balazsfi et al., 2017). We have to note, however, that experimental conditions of the behavior experiments as well as *in vivo* and *in vitro* release experiments of the present study were not entirely identical; the link between them is that all three types of experiments were conducted 8 weeks after ChR2 injection and 20 Hz photostimulation was applied to optogenetically activate MRR neurons. Although we have performed release experiments acutely, it would be interesting to further explore the delayed effect of optogenetic activation of MRR on the release of different transmitters, when the freezing effect is already developed. Nevertheless, a support that *P2rx7*s play a role in aversive memories comes from the literature; Campos et al. has recently found that genetic deletion of *P2rx7*s, as well as intrahippocampal injection of a specific *P2rx7* antagonist counteracted freezing in a contextual fear conditioning paradigm in rodents up to 7 days after the initial training (Campos et al., 2014).

We have used a similar, although not completely identical photostimulation protocol to induce 5-HT release detected radiochemically in perfusate samples of acute MRR and hippocampal slices, derived from virus injected mice. In the MRR OS released 5-HT in a reproducible manner, and its amount was comparable, but less than that evoked by electrical and chemical depolarization. This difference is reasonable as ES activates the whole incoming nerve bundle, whilst OS is more specific, and stimulates only those neurons and their nerve terminals that express the ChR2 protein. This observation indicates the presence of serotonergic nerve terminals in the MRR originating outside the area reached by virus injection consistently with the reciprocal innervation MRR and other brainstem serotonergic nuclei, such as the DR (Chazal and Ralston, 1987).

5-HT release in the MRR evoked by both optical and electrical stimulation was [Ca^2+^]_o_-dependent showing that it is mediated by vesicular exocytosis. Both electrically and optically induced 5-HT efflux was also sensitive to antagonists of ionotropic Glu receptors implying that 5-HT is released by Glu from ChR2 expressing MRR neurons. This is compatible with anatomical studies identifying autapses and recurrent axon collaterals of MRR neurons (e.g., Adell et al., 2002). 5-HT-containing neurons are immunopositive for Glu (Ottersen and Storm-Mathisen, 1984) as well as for phosphate-activated glutaminase (PAG) (Kaneko et al., 1990), a key enzyme involved in Glu biosynthesis, and 5-HT neurons of raphe nuclei express VGluT3 (Gras et al., 2002). Further, to support this assumption, here we show the simultaneous [^3^H]Glu efflux in response to an identical optogenetic stimulation (**Figure 4G**). Because light stimulation-evoked 5-HT efflux was sensitive to TTX, the released 5-HT and Glu are probably originated from distinct nerve terminal populations and 5-HT might also be derived from dendrites and cell bodies (De-Miguel and Nicholls, 2015). It is well known that 5-HT is secreted from the somata and dendrites of neurons in the raphe nuclei in response to the activation of NMDA receptors in a [Ca^2+^]_o_-dependent way (de Kock et al., 2006; Harsing, 2006), and the majority of 5-HT is released in a non-synaptic manner (Harsing, 2006; Vizi et al., 2010). The novelty in our study is the identification of MRR activated by the optogenetic technique as the neuronal subpopulation representing the above features.

We have also tested, whether 5-HT is released in a similar way from MRR terminals of one of the target areas of MRR, the HP. All three stimulation evoked 5-HT efflux, comparable to the responses measured in the MRR; however, in absolute amount optical and electrical stimulation released less 5-HT from HP than in the MRR. This is consistent with a less intensive EYFP labeling found in the HP (**Figure 1**) corresponding to a less dense innervation in the remote target area.

Whilst 5-HT efflux by both optical and electrical stimulation was largely dependent on [Ca^2+^]_o_ in this area as well, subsequent experiments revealed important differences in the two types of transmitter release in the HP. They displayed a different pattern of frequency dependence, indicating that different transmitter pools are mobilized by subpopulations of nerve terminals activated by the two types of stimulation at different frequencies. In addition, whilst ES induced 5-HT release was inhibited by TTX and Glu receptor antagonists, OS induced 5-HT efflux was not sensitive to the above drugs, which indicates that in the HP ChR2 expressing nerve terminals were directly activated by light stimulation and tritium release represents 5-HT efflux from varicosities originating in the MRR. Because ChR2 is permeable to sodium (Schneider et al., 2013), it is reasonable to assume that direct ion flux through the channel is responsible for the local depolarization of the nerve terminal membrane and subsequent initiation of transmitter release in this case. An interesting additional observation is that ES induced 5-HT release in the HP was sensitive to the blockade of ionotropic Glu receptors. A plausible explanation for that is that ES, in contrast to light stimulation, also depolarizes excitatory nerve terminals and releases glutamate, which then acts on ionotropic glutamate receptors and directly or indirectly mobilize 5-HT from nerve terminals originating in the DR. In a previous paper (Varga et al., 2009), a powerful and unexpectedly fast activation of synaptically targeted interneurons by the MR-hippocampal projection was demonstrated, which was mediated by both serotonin (5-HT) and Glu. Therefore, a similar, glutamatergic DR-hippocampal projection, or hippocampal principal neurons could be the source of glutamate releasing 5-HT in the HP.

Midbrain serotonergic neurons are equipped with somatodendritic and nerve terminal inhibitory autoreceptors (Vizi, 2000). These receptors have been widely implicated in the effect of various drugs affecting serotonergic transmission such as serotonin reuptake inhibitor (SSRI) antidepressants and anxiolytic drugs. Whereas activation of somatodendritic autoreceptors decrease the firing rate of the neurons locally, activation of nerve terminal autoreceptors decrease 5-HT release at the target areas, i.e., in the cortex and HP, and both belong to the 5-HT1_B/D_ subtype (Maura et al., 1986, 1993; Maejima et al., 2013). In our experiments sumatriptan, the 5-HT1_B/D_ agonist decreased 5-HT release by electrical, but not by OS, whereas buspirone, the selective 5-HT_1A_ agonist was without effect to either response. The most likely explanation for the lack of modulation of *OS*-induced 5-HT efflux by 5-HT1_B/D_ receptors is that serotonergic neurons expressing 5-HT1_B/D_ receptors at their hippocampal nerve terminals are located outside the MRR, and they are most likely in the DR. This is consistent with the effects reported before using different techniques to stimulate neuronal activity in the DR and measure 5-HT efflux in the HP (Pineyro et al., 1995; Hopwood and Stamford, 2001; Richer et al., 2002). Our data illustrates the suitability of optogenetic method to investigate subpopulation-specific modulation of neurochemical transmission, which were largely unknown until know.

The second objective of the study was to reveal the potential modulation of 5-HT from hippocampal terminals by *P2rx7*. We found that both genetic deletion and pharmacological blockade of *P2rx7*s decreased 5-HT release evoked by OS as well as focal ES of the incoming nerve bundle. These findings indicated that endogenous ATP released by the above stimulation paradigms, promoted the release of 5-HT from serotonergic varicosities through the activation of *P2rx7*s. To support this assumption found that identical stimulation significantly elevated extracellular ATP level in this brain area. Because 5-HT release evoked by both optical and electrical stimulation was decreased by the inhibition of *P2rx7s*, the local control exerted by *P2rx7* activation in the HP extends on the 5-HT nerve terminals from both median raphe neurons and non-median raphe neurons. Furthermore, since 5-HT release evoked by OS was insensitive to TTX, a reasonable assumption is that *P2rx7*s driving the modulation of 5-HT efflux from MRR terminals are expressed on the terminals themselves. Nevertheless, the possibility that 5-HT release is modulated by a compound released from nearby astrocytes in response to the activation of astroglial *P2rx7*s cannot be entirely excluded. Our data showing the *P2rx7*-mediated modulation of [^3^H]5-HT release are not contradictory with previous findings reporting elevation in endogenous 5-HT level and inhibition of 5-HT transporters in case of genetic deletion and the pharmacological blockade of *P2rx7*s (Csolle et al., 2013b; Lord et al., 2014). These changes might be independent and compensatory changes as well, pointing to the complex regulation of serotonergic transmission by *P2rx7*s. The regulation of 5-HT efflux by *P2rx7s* might also gain significance in psychiatric disorders as recent studies pointed out the alleviation of depression- and schizophrenia-like behaviors in rodent animal models by the inhibition of P2rx7s (Csolle et al., 2013a,b; Kovanyi et al., 2016).

Finally, we have verified our findings obtained in *in vitro* slice experiments *in vivo*, to detect the parallel release of neurotransmitters to a similar OS. We demonstrate here that 5-HT and Glu are released simultaneously by 20Hz OS, whereas GABA was not elevated by a detectable level by this stimulus. On the other hand, all three neurotransmitters responded equally well to K^+^ depolarization, which is not restricted to ChR2 expressing neurons.

In contrast to 20 Hz stimulation, 50 Hz theta bursts lead to significant elevation in the level of GABA, but did not release either 5-HT or Glu. This result illustrates that different patterns of neuronal activity in the MRR results in distinct patterns of transmitter efflux. Since the second OS was relatively closed to the run-down of the first response, one might assume, that the releasable pool of transmitters have not been replenished yet at this time point. However, this was not the case, because if the order of stimulations was replaced, 50 Hz was still ineffective to release either 5-HT or Glu (Supplementary Figure 3).

## Conclusion

Optogenetic activation is a feasible method to selectively release and directly detect transmitters from an identified pathway *in vitro* and *in vivo* by propagating Na^+^ channel activity and subsequent Ca^2+^-dependent exocytosis. Our results show that in the MRR, 5-HT and Glu are both released from ChR2 expressing neurons of MRR and 5-HT release is the result of ionotropic Glu receptor activation. In the HP, 5-HT is released from MRR terminals independently from glutamatergic transmission and is not subject to neuromodulation by presynaptic 5-HT1_B/D_ autoreceptors. In contrast hippocampal 5-HT release from both MRR and non-MRR terminals is subject to neuromodulation by endogenous activation of *P2rx7*.

## Author Contributions

BS, TF, and JH conceptualized the project and obtained funding. FG performed and analyzed *in vitro* release experiments. DB and DZ performed virus injection, behavior and microdialysis experiments, MB conducted HPLC analyses and KD carried out confocal microscopy. FG, MB, DZ, TF, and BS wrote the paper.

## Conflict of Interest Statement

The authors declare that the research was conducted in the absence of any commercial or financial relationships that could be construed as a potential conflict of interest.

## Abbreviations

5-HT: 5-hydroxytryptamine (or serotonin)
AAV: adeno associated virus vector
AMPA: α-amino-3-hydroxy-5-methyl-4-isoazolepropionic acid
ANOVA: analysis of variance
ATP: adenosine triphosphate
AUC: area under the curve
BUSP: buspirone
ChR2: Channelrhodopsin-2
CNQX: 6-cyano-7-nitroquinoxaline-2,3-dione-disodium
DL-AP-5: DL-2-amino-5-phosphonopentanoic acid
DNA: deoxyribonucleic acid
DR: dorsal raphe
ES: electrical stimulation
GABA: γ-amino-butyric acid
Glu: glutamate
HP: Hippocampus
HPLC: high-performance liquid chromatography
KO: knockout
MR: median raphe nuclei
MRR: median raphe region
NMDA: *N*-methyl-D-aspartate
OS: optical stimulation, *P2rx7*, P2X7 receptor
PAG: phosphate-activated glutaminase
PBS: phosphate buffered saline
SEM: standard error of the mean
SUMA: sumatriptan
TTX: tetrodotoxin
VGluT3: vesicular glutamate transporter 3
WT: wild-type
