# Supplementary material for: Regulation of Hippocampal 5-HT Release by P2X7 Receptors in Response to Optogenetic Stimulation of Median Raphe Terminals of Mice
Source: Front Mol Neurosci. 2017 Oct 12;10:325. doi: 10.3389/fnmol.2017.00325 (PMC5643475; doi:10.3389/fnmol.2017.00325)
Supplement: Supplementary file 1 [file Data_Sheet_1.pdf]

Supplementary Material

Regulation of hippocampal 5-HT release by P2X7 receptors in response to optogenetic stimulation of median raphe terminals of mice

Flóra Gölöncsér, Mária Baranyi, Diána Balázsfi, Kornél Demeter, József Haller, Tamás F. F. Freund, Dóra Zelena and Beáta Sperlágh*

*** Correspondence:** Corresponding Author: sperlagh@koki.hu

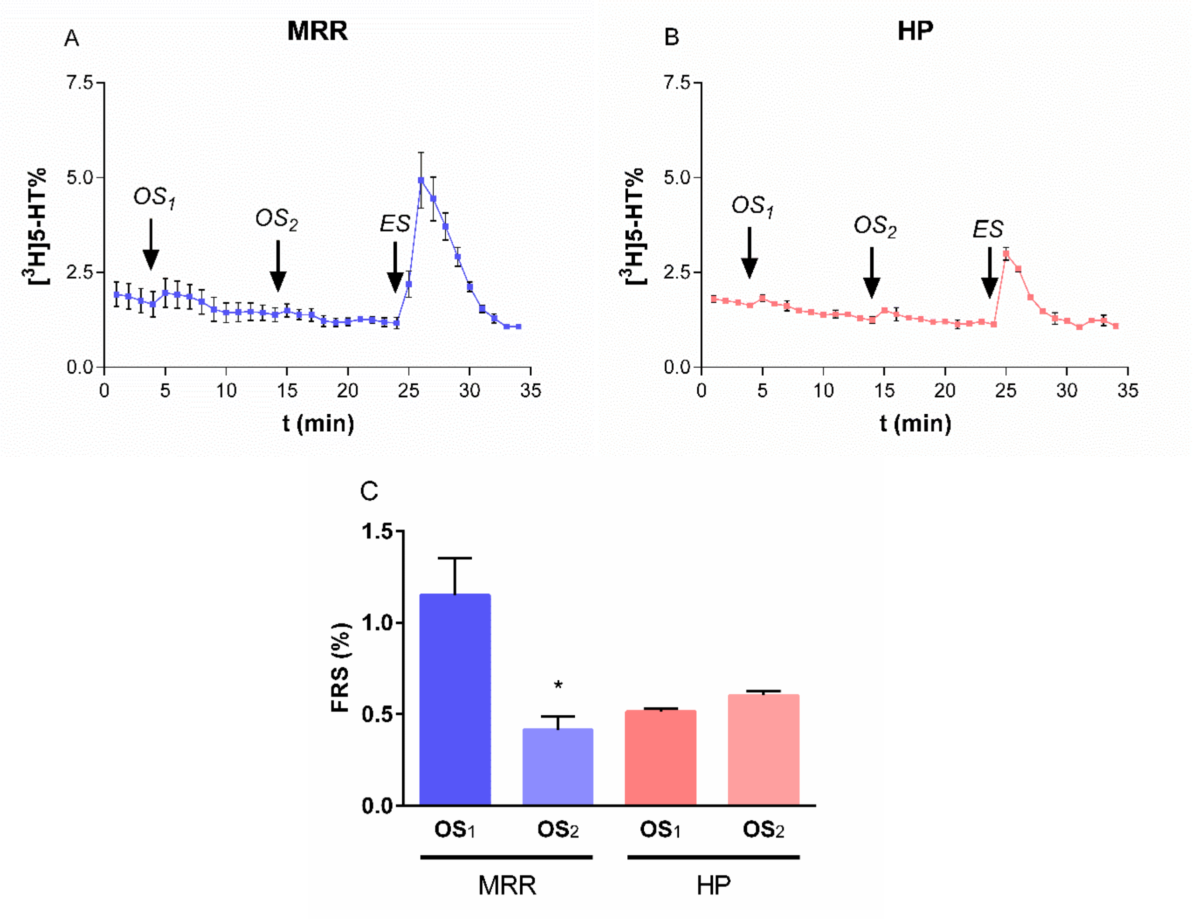

Supplementary Figure 1. Optical stimulation results in negligible release of [^{3}H]5-HT in the MRR and HP slices *in vitro* 4 weeks after virus injection. At first, we used animals 4 weeks after virus injection in the release experiments using the protocol of "Experiment 2". However, optical stimulation resulted in only a slight, insignificant elevation of tritium efflux in the MRR and HP under these conditions. Optical and electrical stimulation have been made on 50Hz. The tritium content in the perfusate samples (**A, B**) was expressed as fractional release (FR%) and is shown as a function of time.

Curve shows the mean ± SEM of 2-5 identical experiments. This can be explained by observations showing that promoters can take over an extended time to generate sufficient expression at the nerve terminal level. As time passes, ChR2 diffuses throughout the cell and populate the membrane of a distal processes. It may take one to two months to find an adequate expression in the terminals of axons. Panel (**C**) shows the net tritium efflux evoked by the optical stimulation period (OS_1, OS_2) in the MRR and HP slices. Results were analyzed by two-way ANOVA, followed by Scheffe test, $^{*}p < 0.05$ OS_1 vs OS_2 in MRR, n = 5.

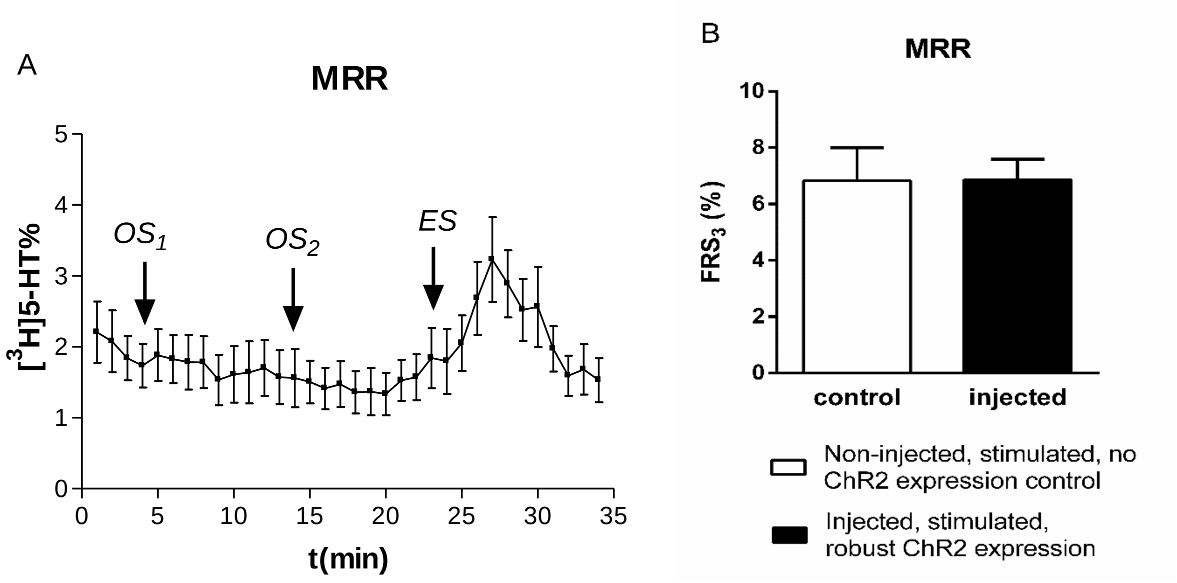

Supplementary Figure 2. Optical stimulation of MRR derived from control, naïve mice did not elicit any increase in tritium efflux, excluding any non-specific effects of optical stimulation. We used the protocol of Experiment 2. Optical and electrical stimulation have been made on 50Hz. The tritium content in the perfusate samples (**A**) was expressed as fractional release (FR%) and is shown as a function of time. Curve shows the mean ± SEM of 4 identical experiments. Graph in panel (**B**) represents the comparison of electrically evoked [^{3}H]5-HT release of control, naïve mice and rAAV-injected, stimulated mice (see in the article, Figure 4C). There was no significant difference among the two groups. The results are expressed as the net release of tritium evoked by the electrical stimulation period (*FRS_3*, %). Results were analyzed by one-way ANOVA.

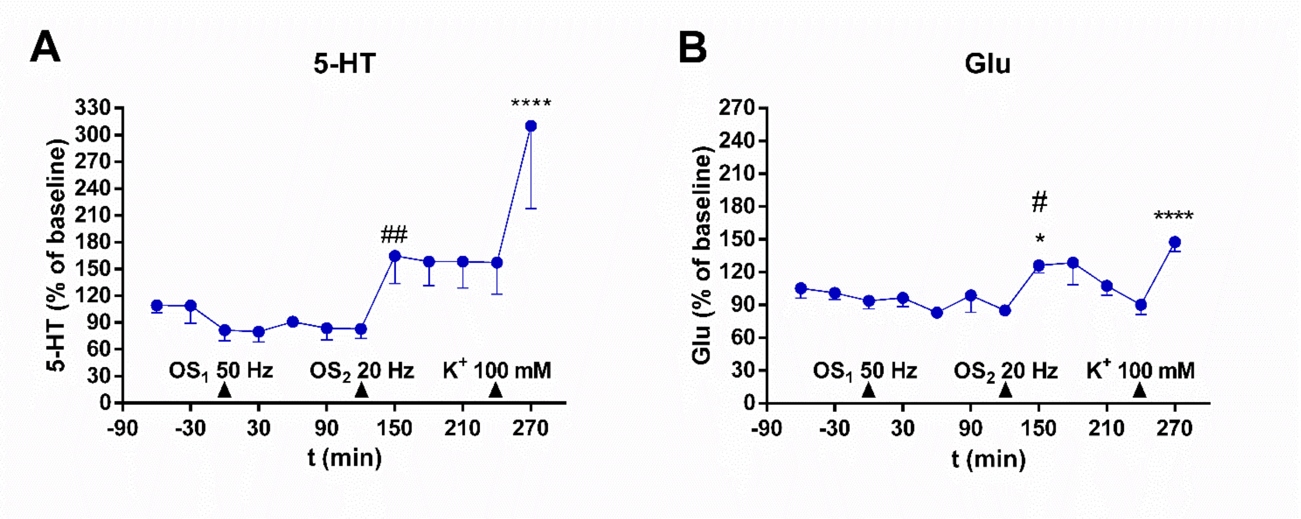

Supplementary Figure 3. *In vivo* light stimulation with 50 Hz did not increase extracellular 5-HT and Glu levels in MMR, even if it was applied as a first depolarization stimulus. Arrows indicate the time of different type of light and chemical stimulations. Data are expressed as % of baseline as means ± SEM (n = 3 per group). Results were analyzed by two-way factorial ANOVA, followed by Fischer LSD *post hoc* test. Asterisks indicate significant differences from baseline, $^{*}p < 0.05$, $^{****}p < 0.0001$. Hash marks indicate significant differences between the effect of two different light stimuli, $^{\#}p < 0.05$, $^{\#\#}p < 0.01$.
